# A Novel Filtering Mutualism between a Sponge Host and Its Endosymbiotic Bivalves

**DOI:** 10.1371/journal.pone.0108885

**Published:** 2014-10-20

**Authors:** Remi Tsubaki, Makoto Kato

**Affiliations:** Graduate school of Human and Environment Studies, Kyoto University, Sakyo, Kyoto, Japan; Rosalind Franklin University, United States of America

## Abstract

Sponges, porous filter-feeding organisms consisting of vast canal systems, provide unique substrates for diverse symbiotic organisms. The *Spongia* (*Spongia*) sp. massive sponge is obligately inhabited by the host-specific endosymbiotic bivalve *Vulsella vulsella*, which benefits from this symbiosis by receiving protection from predators. However, whether the host sponge gains any benefit from this association is unclear. Considering that the bivalves exhale filtered water into the sponge body rather than the ambient environment, the sponge is hypothesized to utilize water exhaled by the bivalves to circulate water around its body more efficiently. We tested this hypothesis by observing the sponge aquiferous structure and comparing the pumping rates of sponges and bivalves. Observations of water currents and the sponge aquiferous structure revealed that the sponge had a unique canal system enabling it to inhale water exhaled from bivalves, indicating that the host sponge adapted morphologically to receive water from the bivalves. In addition, the volume of water circulating in the sponge body was dramatically increased by the water exhaled from bivalves. Therefore, this sponge-bivalve association can be regarded as a novel mutualism in which two filter-feeding symbionts promote mutual filtering rates. This symbiotic association should be called a “filtering mutualism”.

## Introduction

**Figure 1:**
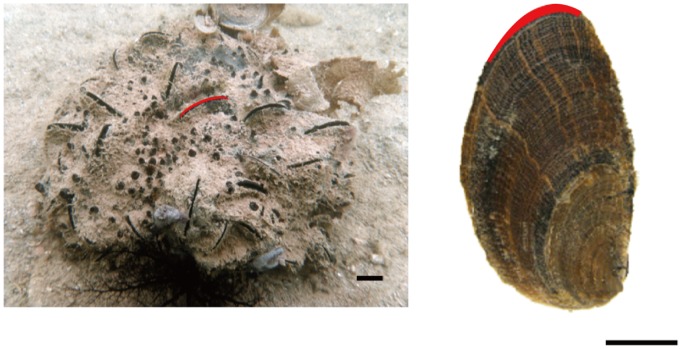
Photograph of *Spongia* sp. *in situ* and the shell of *Vulsella vulsella*. The red line of the sponge corresponds to that of the shell; scale bars = 10 mm.

Sponges are multicellular, porous, filter-feeding organisms with vast canal systems [1], and they provide unique substrates for diverse organisms [2]. One group of sponge-associated macro-organisms consists of bivalves belonging to genus *Vulsella,* which are known to inhabit specific sponge species [3], [4]. These bivalves are so highly specialized for sponge-embedded life that they have lost the byssus by which bivalves attach to hard substrate; their shells are completely embedded in the sponge and only a short length of ventral commissure communicates with the external environment (Fig. 1). The association between *Vulsella vulsella* and its host sponge is believed to be mutualistic, with the bivalve serving as an endoskeleton for the sponge, and the sponge protecting the bivalve from predators via inaccessibility and noxious secretions [5]. In addition, *V. vulsella* was hypothesized to exploit the host sponge as a sink for its exhalant current and to take advantage of the passive flow induced by the sponge [6]. However, considering the obligate association between the sponge and the bivalve and the high density of symbiotic bivalves in a sponge [4], it is natural to assume that the sponge utilizes the strong excurrent jet created by the bivalves as a water-circulating pump, contrary to the traditional view.

Suspension feeders depend on surrounding or self-created water currents for feeding, respiration and dispersal of gametes. Sponges create water currents by flapping the flagella of their choanocyte cells and ingest small particles from the incurrent water. A massive sponge may find it difficult to circulate enough water through its whole body without paying a considerable cost to create a large number of choanocyte chambers and move flagella. Recently, the energetic cost of pumping in the sponge *Negombata magnifica* was estimated at up to 30% of the total metabolism, which is much higher than previously believed [7]. The weakness and high cost of the choanocyte flagella-based pumping system in sponges suggests that the strong gill cilia-based pumping system of bivalves would be highly desired by sponges.

In this study, we observed water currents in the sponge-bivalve symbiotic system in detail and tested the hypothesis that the host sponge *Spongia* (*Spongia*) sp. (identified as undescribed species, see details File S1 for details on morphology) utilizes the excurrent water from *V. vulsella* to circulate water in the whole sponge body via directly observing water currents in the sponge aquiferous system with tracking dye and by comparing pumping rates between the sponge and the bivalve by measuring dye flow.

## Materials and Methods

*Spongia* (*Spongia*) sp. individuals that contained many *Vulsella vulsella* individuals were collected in Haneji Inland Sea at the northern part of Okinawa Island, Japan (26°38′55″ N, 128°0′30″ E). In this location, no specific permissions were required and both *Spongia* (*Spongia*) sp. and *V. vulsella* are not endangered or protected species. We conducted samplings by snorkeling at a tidal flat situated in Haneji Inland Sea. Surface seawater temperature was varied among 19 and 30°C, inferred from coastal observations at Sesoko Station, Tropical Biosphere Research Center University of Ryukyus (26°38′ N, 127°51′ E). The sea bottom is mainly composed of sand and gravel, is muddy, and gradually deepens. All sample collection was conducted at the depth up to 1 m during lowest water levels of the spring tide. *Spongia* (*Spongia*) sp. is common species in our study site and all *Spongia* (*Spongia*) sp. we collected contained *V. vulsella* [4].

At least 1 h before any experiment, a single *Spongia* (*Spongia*) sp. individual was transferred to an aquarium and acclimated to the experimental conditions.

First, a solution of yellow fluorescent dye (Wako Pure Chemical Industries, Ltd., Osaka, Japan) was injected into the exposed slits of all *V. vulsella* bivalves embedded in three *Spongia* (*Spongia*) sp. individuals. By tracking the dye flow, we noted where the dyed water was exhaled. After observing the dyed water currents, resin-cast replicas of the aquiferous systems were obtained for each sponge by injecting liquid resin into an osculum as described by [8]. A plastic resin (Batson’s #17, Polysciences Ind., Warrington, PA) was injected in vivo through the oscula, upstream of the water flow, until it leaked out through inhalant pores at the sponge surface. To make the plastic resin more visible, the resin was dyed red. Approximately 12 h after resin injection, the specimens were immersed in a 20% sodium hypochlorite solution for 24 h to remove all organic matter. After careful washing, fragments of the casts were mounted on stubs and observed by stereoscopic microscopy and scanning electron microscopy (VE-7800, Keyence, Tokyo, Japan).

To compare the pumping rate of host sponge and bivalves, we estimated the pumping rate of whole sponge containing bivalves and bivalves alone. We did not conducted experiment on sponge without bivalves because the removal of embedded bivalves severely damage sponge body. The pumping rates of the sponges and bivalves were determined by videotaping the movement of dye fronts in the excurrent regions, as described by [9]. A video camera was set up directly in front of the excurrent water flow, and small puffs of a concentrated fluorescent dye solution, held in a dropper, were released into the ambient water. Video images of dye movement were checked frame by frame and dye speed was calculated as the distance travelled by the sharp leading edge of the puffs perpendicular to the plane of the osculum, over as many frames as possible, divided by the time in which it travelled that distance. The interval between frames was 1/30 s. The excurrent speeds were measured for 29 oscula from four sponge individuals, and their diameters were also determined. The average volume of excurrent water per osculum was estimated by multiplying the average excurrent speed by the mean cross-sectional area of the sponge oscula. The pumping rate of the whole sponge was obtained by multiplying the number of oscula in an individual sponge by the obtained average pumping rate per osculum. To estimate the pumping rates of the bivalves, we dissected the sponges, removed all bivalves and transferred them to aquaria as soon as possible. For the bivalves in aquaria, the speed of exhaled water was estimated using the same procedure that was used for the sponges. The excurrent speed was measured for 43 specimens from five sponges. We measured the cross-sectional area of the excurrent region, and the pumping rate of a bivalve was obtained by multiplying the cross-sectional area of the bivalve’s excurrent region by the excurrent speed. As the water volume exhaled per second is strongly correlated with the bivalve’s shell size, we measured the shell height of the tested bivalves and estimated a regression equation to obtain the water volume exhaled per second by each bivalve (see Results). The total water volume exhaled by all the embedded bivalves in a sponge individual was obtained by adding together the water volume exhaled by all bivalves embedded in that sponge. By comparing the water volume exhaled by the host sponge with the volume exhaled by symbiotic bivalves, we estimated the extent to which the sponges depend on the bivalves’ excurrent water.

## Results

### (a) Direct observations of dye movement and the sponge aquiferous system

**Figure 2:**
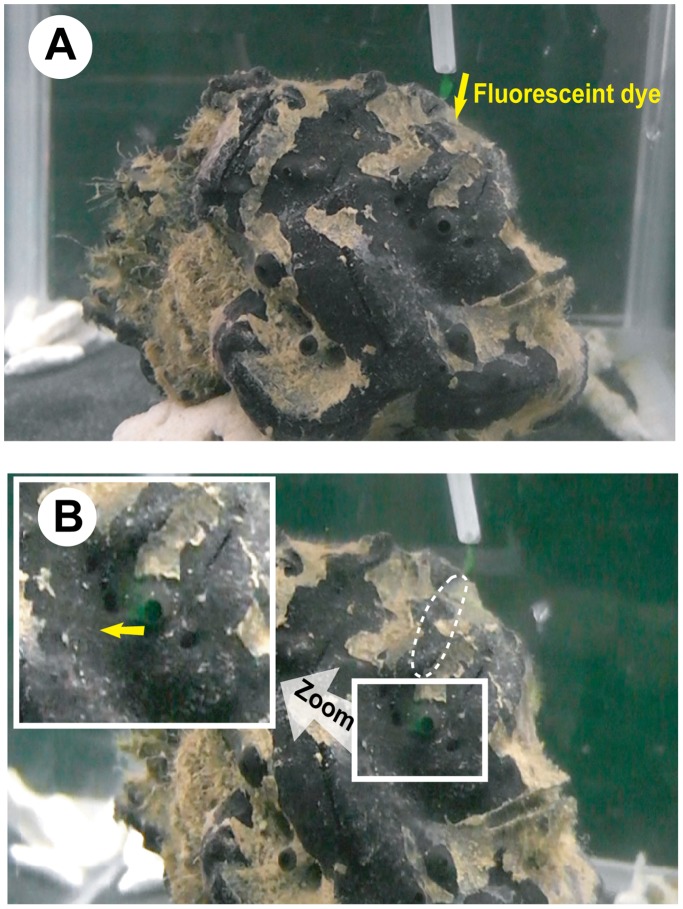
Photographs of the dye movement experiment. A. Fluorescent dye dropped into the inhalant area of the bivalves. B. Dropped fluorescent dye first inhaled into the bivalve’s body, and then exhaled from specific sponge oscula.

The three examined *Spongia* (*Spongia*) sp. individuals harbored 56 *Vulsella vulsella* (30, 13 and 13, respectively). The fluorescent dye was successfully traced for 41 bivalves, while the others were inactive during the experimental period. In each successful case, the dropped dye was first inhaled into the bivalve’s body, and then exhaled from one or several nearby host oscula (Fig. 2; Table 1). The results indicate that the host sponge’s canal structure enabled it to absorb the bivalve’s exhalant water into its aquiferous system.

**Table 1:** Summary of direct observations of movement of fluorescent dye dropped at the inhalant region of bivalves.

ID	Dry weight (g)	Volume (ml)	No. ofoscula	No. ofbivalves	No. of bivalves thatabsorbed dye	No. of oscula thatexhaled dye	No. of oscula connected withmore than one bivalve
1	6.61	95	50	30	19	26	6
2	6.91	163	69	13	10	11	4
3	3.32	110	28	13	12	7	4
Total	16.84	368	147	56	41	44	14

**Figure 3:**
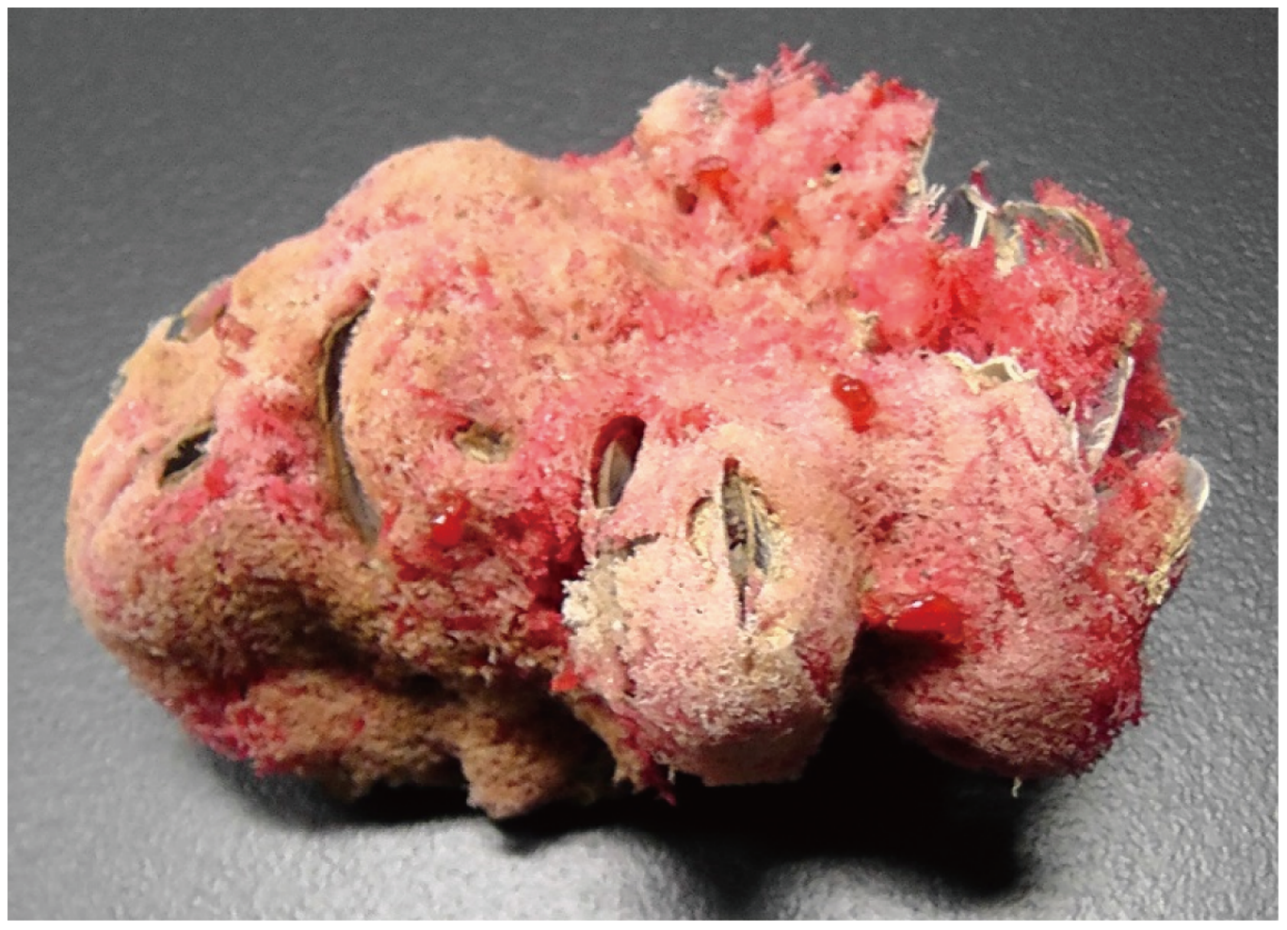
The whole plastic resin casting of the aquiferous system of *Spongia* (*Spongia*) sp.

**Figure 4:**
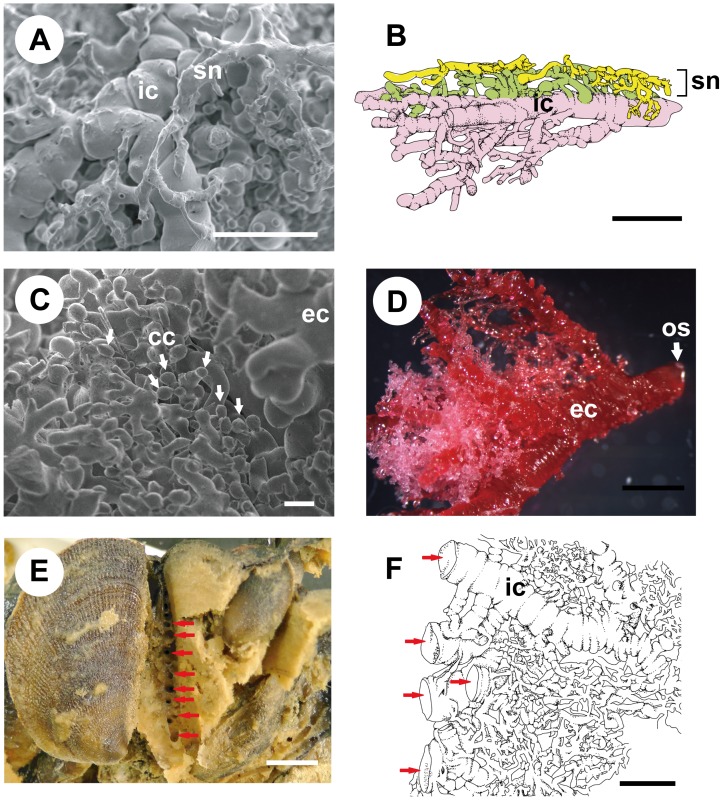
SEM micrograph and drawing of canal replica of *Spongia* (*Spongia*) sp. A. Scanning electron microscope (SEM) micrograph of sponge surface area showing the superficial network of incurrent canals (sn) connected with main incurrent ducts (ic); scale bar = 500 µm. B. Side-view drawing of the sponge surface incurrent area; scale bar 500 = µm. C. SEM micrograph showing a clump of choanocyte chambers (cc, arrows) and excurrent canals (ec) forming an anastomosed framework; scale bar = 100 µm. D. SEM micrograph of anastomosed excurrent canals leading to an osculum (os). E. Photograph of a dissected sponge, in which a bivalve was unfastened and moved to the left so that the opening of the excurrent canal can be seen (arrows). F. Side-view drawing of the incurrent system facing a bivalve’s exhalant area. Arrows correspond to the holes in Fig. 4E.

Observation of the resin replicas showed that the aquiferous system of *Spongia* (*Spongia*) sp. is composed of a dense arrangement of dendritic canals (Figs. 3, 4). The outermost part of the casts consisted of a network of anastomosized canals throughout the surface area of the entire sponge (Fig. 4A, B). The superficial network was formed by inhalant canals and connected to the main incurrent ducts that were approximately four to five times larger in diameter than the superficial incurrent canals. These ducts ramified and formed anastomoses, resulting in a three-dimensional web. The incurrent ducts extended throughout the whole sponge body to distribute water to the various sponge regions. Choanocyte chambers, which connected to the excurrent canals, were found almost everywhere in the choanosome (Fig. 4C). Groups of choanocyte chambers were drained by small canals that merged into the excurrent canals. The anastomosed excurrent canals converged on oscular ducts leading to oscula (Fig. 4D).

**Figure 5:**
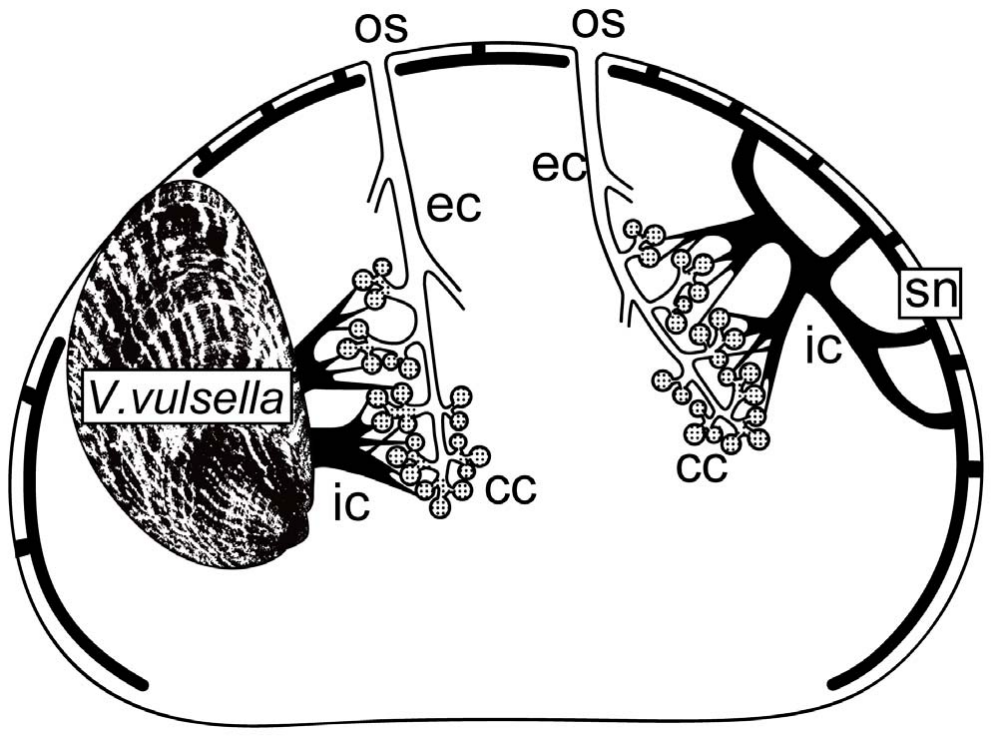
Diagram showing the architecture of the aquiferous system of *Spongia* (*Spongia*) sp. *Black canals* = incurrent; *white canals* = excurrent; *dotted areas* = choanocyte chambers.

The aquiferous system of sponge surface area that faced a bivalve’s exhalant jet was markedly different from that of sponge surface area that faced the ambient environment. When dissecting the sponge, we could detect several large holes facing a bivalve’s exhalant area, the size of which ranged from 0.6 to 2.8 mm (Fig. 4E). Based on direct observation of dye movement, water inhaled by bivalves was drained into these holes. The replica of the aquiferous system around this area showed that the large incurrent ducts leading to the holes facing bivalves penetrated into the sponge body, ramifying into small canals, which branched into twigs with choanocyte chambers on their tips (Fig. 4F). Based on these observations, the architecture of the main canals of the sponge is summarized in Fig. 5.

### (b) Pumping rates

**Figure 6:**
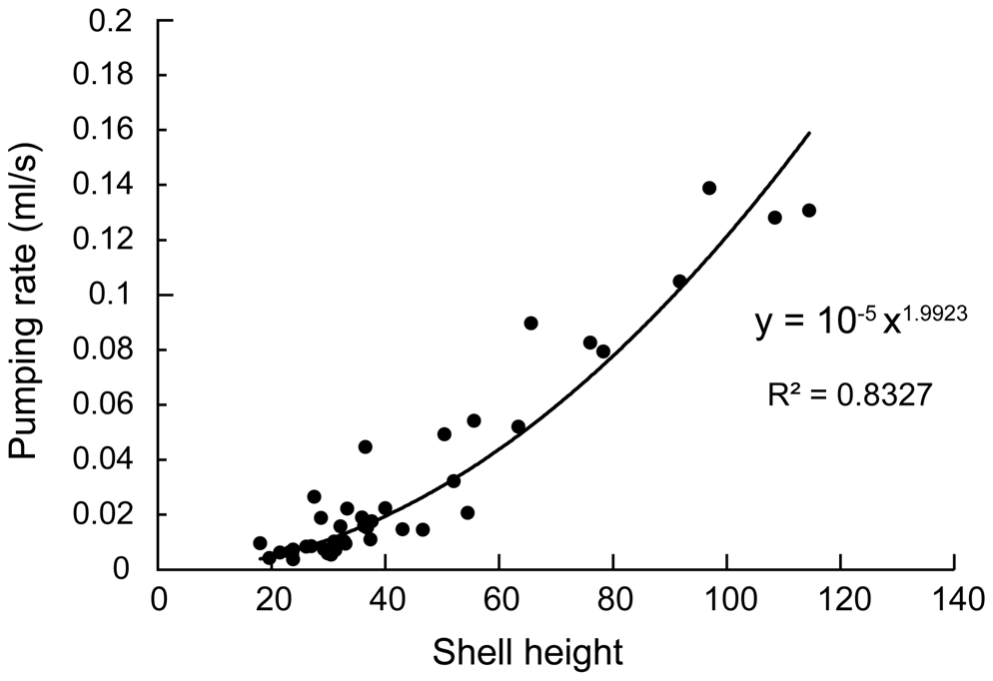
Correlation between shell length and pumping rate of *V. vulsella* (N = 43).

The pumping rate of an osculum was estimated at 0.0087±0.0019 ml/s (mean ± S.E.). Based on this estimation, the entire water volume passing through a sponge body was calculated at 0.251–0.598 ml/s (Table 2). The exhaled water volume of the bivalves was obtained by the following equation determined by our experiments: the water volume exhaled by a bivalve per second = 10-^5^ *H* ^1.993^, where *H* is the shell height (Fig. 6).

**Table 2:** Pumping rates of sponge and bivalves.

					Pumping rate of			
Specimen ID	Dryweight (g)	Volume(ml)	Density(g/ml)	No. ofbivalves	sponge including bivalves(ml/sec)	sponge including bivalves(ml/sec)	sponge(ml/sec/g dry weight)	Dependence of sponge onbivalves' excurrent water
1	6.4	1.6	0.060	56	0.254	0.143	0.039	0.57
2	4.9	85	0.058	39	0.269	0.162	0.055	0.6
3	10.0	140	0.071	36	0.269	0.219	0.027	0.82
4	10.8	180	0.060	90	0.563	0.249	0.052	0.44
5	8.38	214	0.039	63	0.598	0.299	0.071	0.50
6	6.61	95	0.070	27	0.260	0.097	0.039	0.37
7	8.75	127	0.069	41	0.286	0.118	0.033	0.41
8	13.5	245	0.055	29	0.572	0.169	0.042	0.30
Mean			0.054					0.50
S.D.			0.022					0.16

Based on these estimations, the proportion of the pumping rate of *V. vulsella* to that of *Spongia* sp. was 50.2±16.2% (mean ± S.D, N = 8; range: 29.6–81.7%; Table 2). This high proportion indicates that the host sponge heavily depends on the excurrent water from the symbiotic bivalves.

## Discussion

Direct observations of dye flow and replicas of the sponge aquiferous system suggest that *Spongia* (*Spongia*) sp. has a unique canal system that enables the sponge to utilize the water exhaled from *Vulsella vulsella* (Fig. 4). Considering that *V. vulsella* passively becomes embedded into the sponge due to sponge growth, as the bivalve has no ability to penetrate into the sponge body, the sponge must construct incurrent canal systems to inhale the bivalve’s excurrent water efficiently when encrusting its excurrent region. Incurrent water from the bivalve would be quite beneficial in distributing water to the inner part of the sponge body. Remodelling of aquiferous systems occurs easily in sponges [1], [10]. The adaptive canal system of *Spongia* (*Spongia*) sp. is formed by the re-plumbing of incurrent and excurrent canals through intimate interaction between the sponge and the bivalve.

The estimated pumping rates of the sponge and the bivalve indicate that the water volume circulating around the host sponge body was dramatically increased by the bivalve’s excurrent water. To save the high cost of pumping activity, the thin-walled sponge *Aphrocallites vastus* was reported to enhance flow through its body by using passive flow induced by the ambient current [11]. Compared to ambient water currents, the utilization of currents from symbiotic bivalves is more beneficial because bivalves continuously exhale water directly into the sponge aquiferous system. The increment of inhalant water from the bivalves’ exhalant jets has several benefits for the sponges: e.g. the accelerated excurrent speed prevents the sponge from re-inhaling already filtered water, sponges gain water flow passively without using energy to move flagellated cells and access to a potential food supply. The two former benefits are probably true for the bivalve as well, as the sponges also generate water flow on their own. From the perspective of food supply, the amount of available food that remains in water exhaled by the bivalves is important. Some sponges are known to have different preferences in particle size; most species preferred picoplankton (<2 µm) to nanoplankton (2–20 µm) [12]–[14]. On the other hand, many bivalves are reported to have the ability to uptake particles of a much broader size range, although the retention efficiency of bivalves is known to decrease with the decrease in particle size (as reviewed in [15]). In any case, the retention efficiency of particles <1 µm in size by all bivalves previously studied was less than 50%, which suggests that based on the differences in their effective grazing rates on small particles, food partitioning may occur between the host sponge and the bivalves. Additionally, the absolute particle amount inhaled by the sponge body is increased by the bivalves’ pumping activity due to the vast amount of the bivalves’ excurrent water, which contains large amounts of uneaten small particles. An oyster *Ostrea permollis* is also known to be embedded in a specific sponge host *Stelletta grubii* [16], [17]. As *O. permollis* also reported to exhale water to sponge inner body, the host sponge *S. grubii* might utilize the oyster as water circulating pump as indicated in the relationship between *Spongia* (*Spongia*) sp. and *V. vulsella*. It is intriguing that the similar sponge-dwelling habit evolved independently in two lineage of bivalves.

In the marine ecosystem, many sessile benthic invertebrates are utilized as substrate by diverse filter-feeding organisms [18]. Because both the hosts and symbionts are filter feeders, they are potentially antagonistic, competing for common suspended organic particles. However, this sponge-bivalve association can be regarded as a novel mutualism in which two species of filter-feeding symbionts promote mutual filtering rates, which should be called “filtering mutualism”. Considering that most symbionts that live in marine sessile organisms with skeletons are filter feeders that either utilize ambient currents or actively create currents to move water in and out of their bodies, the novel mutualism in this case is largely owed to the porosity and regenerability of the canal system of the sponge.

## Supporting Information

File S1 **Short description of** ***Spongia*** **(** ***Spongia*** **) sp.**
(DOCX)
