# Supplementary material for: A Novel Filtering Mutualism between a Sponge Host and Its Endosymbiotic Bivalves
Source: PLoS One. 2014 Oct 20;9(10):e108885. doi: 10.1371/journal.pone.0108885 (PMC4203694; doi:10.1371/journal.pone.0108885)
Supplement: File S1 — Short description of Spongia ( Spongia ) sp. (DOCX) [file pone.0108885.s001.docx]

***Spongia* (*Spongia*) sp.**

**Material examined**

Eight specimens examined were collected by snorkeling at a subtidal flat situated in Haneji Inland Sea at the northern part of Okinawa Island, Japan (26°38’55” N, 128°0’30” E).

**Description**

A massive species, forming a roughly globular shape with numerous small oscules (1 to 7 mm in diameter) at the top of short conules on its surface. The texture of this sponge is moderately firm and compressive. This species is black externally, and the internal matrix and fibers are rusty orange. The choanosome is porous with fine canals (less than ~1 mm) rather than wide canals and subdermal lacunae are present just below the ectopinacoderm. Surface membrane has abundant pigment granules. The examined specimens are 15 to 20 cm in width and 10 to 20 cm in height and all the specimens the authors collected so far contained *Vulsella vulsella* bivalves.

In this species, spicules are absent and the body is supported by sponging fibers. Primary fibers are rarely cored by foreign debris. Diameter of primary fibers is 30.0–(32.2)–37.5 µm. Secondary fibers are uncored. Diameter of secondary fibers is 12.5–(18.4)–22.5 µm. Mesh structure variable, subcircular, triangular, rectangular to polygonal. Mesh size is variable, up to 280 µm. The skeleton is mainly comprised of secondary and thertiary fibers, which are uniform in diameter. There is no special dermal skeleton. The skeleton and choanosome collagen contained no foreign debris.


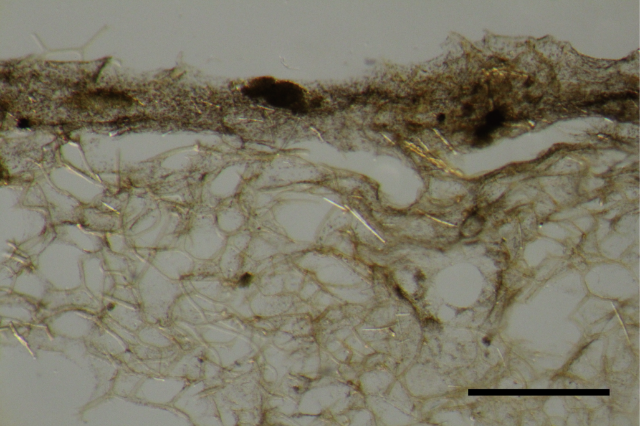

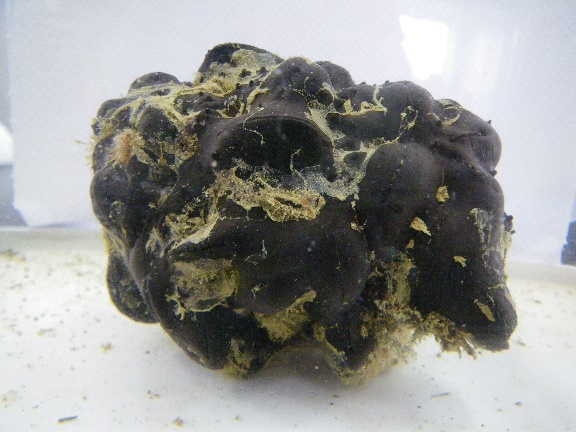

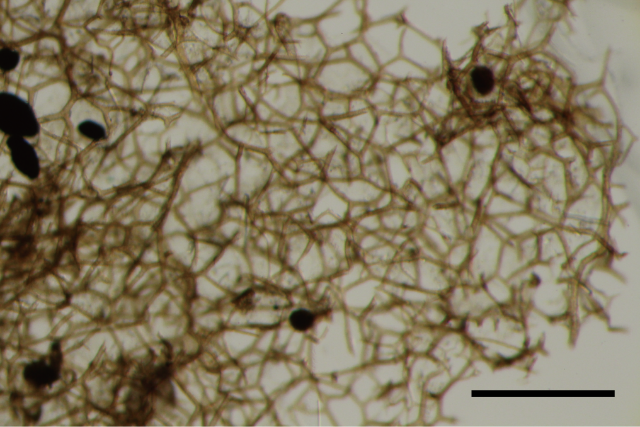


B

C

A

D


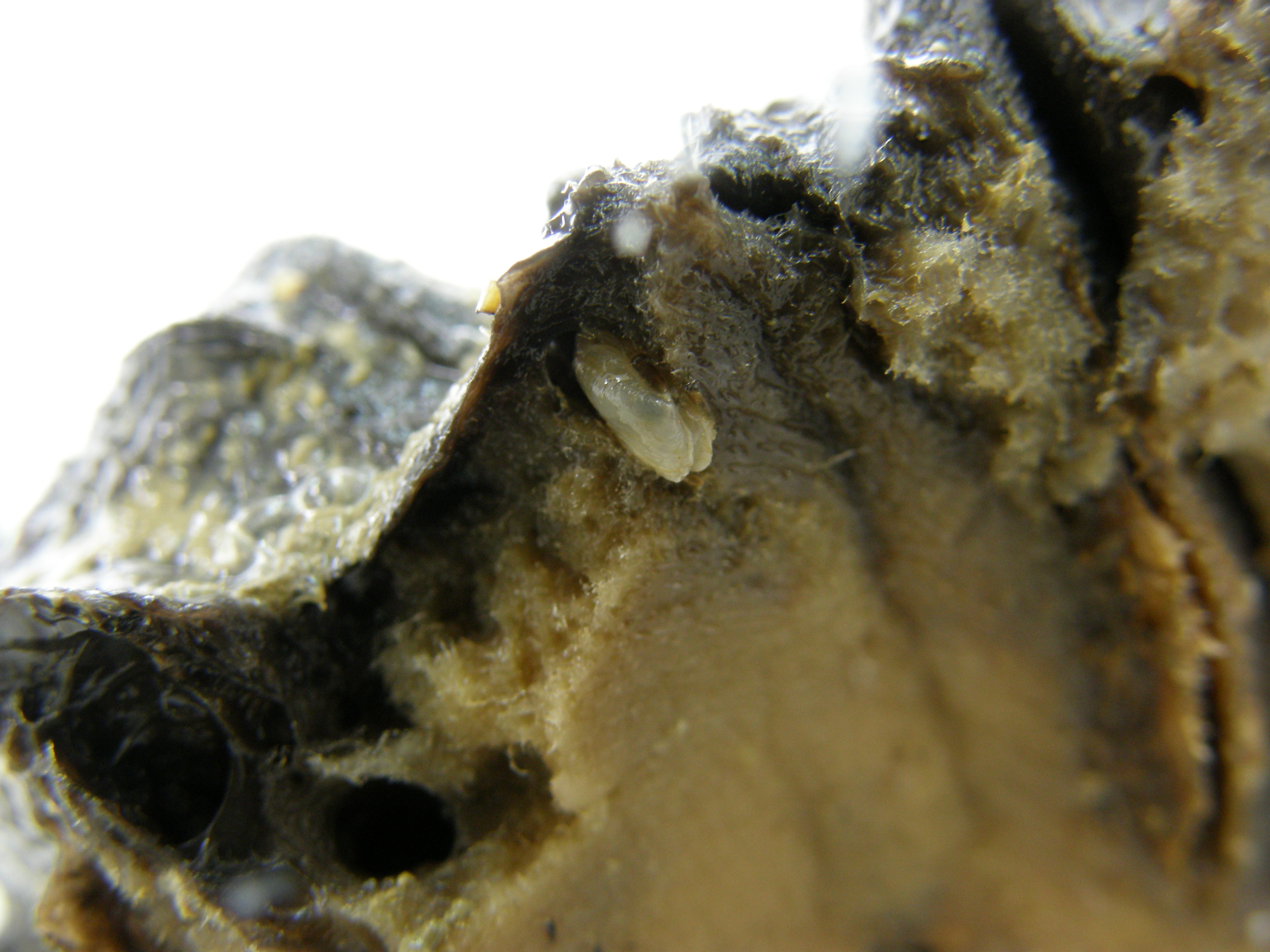


*Spongia* (*Spongia*) sp. A. whole body, B. partial cross section, and details of fiber skeleton of C. surface area and D. inner parts. C and D, scales 500 μm.
